# Interpretation of DAS28 and its components in the assessment of inflammatory and non-inflammatory aspects of rheumatoid arthritis

**DOI:** 10.1186/s41927-018-0016-9

**Published:** 2018-03-23

**Authors:** Daniel F. McWilliams, Patrick D. W. Kiely, Adam Young, Nalinie Joharatnam, Deborah Wilson, David A. Walsh

**Affiliations:** 1 Arthritis Research UK Pain Centre, NIHR Nottingham Biomedical Research Centre & Division of Rheumatology Orthopaedics and Dermatology, University of Nottingham Clinical Sciences Building, City Hospital, Nottingham, NG5 1PB UK; 2 Department of Rheumatology, St Georges Healthcare NHS Trust, London, UK; 3 University of West Hertfordshire, Watford, UK; 4 Department of Rheumatology, Sherwood Forest Hospitals NHS Foundation Trust, Sutton-in-Ashfield, UK

## Abstract

**Background:**

DAS28 is interpreted as the inflammatory disease activity of RA. Non-inflammatory pain mechanisms can confound assessment. We aimed to examine the use of DAS28 components or DAS28-derived measures that have been published as indices of non-inflammatory pain mechanisms, to inform interpretation of disease activity.

**Methods:**

Data were used from multiple observational epidemiology studies of people with RA. Statistical characteristics of DAS28 components and derived indices were assessed using baseline and follow up data from British Society for Rheumatology Biologics Registry participants (1) commencing anti-TNF therapy (*n* = 10,813), or (2) changing between non-biologic DMARDs (*n* = 2992), (3) Early Rheumatoid Arthritis Network participants (*n* = 813), and (4) participants in a cross-sectional study exploring fibromyalgia and pain thresholds (*n* = 45). Repeatability was tested in 34 patients with active RA. Derived indices were the proportion of DAS28 attributable to patient-reported components (DAS28-P), tender-swollen difference and tender:swollen ratio. Pressure pain detection threshold (PPT) was used as an index of pain sensitisation.

**Results:**

DAS28, tender joint count, visual analogue scale, DAS28-P, tender-swollen difference and tender:swollen ratio were more strongly associated with pain, PPT and fibromyalgia status than were swollen joint count or erythrocyte sedimentation rate. DAS28-P, tender-swollen difference and tender:swollen ratio better predicted pain over 1 year than did DAS28 or its individual components.

**Conclusions:**

DAS28 is strongly associated both with inflammation and with patient-reported outcomes. DAS28-derived indices such as tender-swollen difference are associated with non-inflammatory pain mechanisms, can predict future pain and should inform how DAS28 is interpreted as an index of inflammatory disease activity in RA.

## Background

The 28 joint disease activity score incorporating erythrocyte sedimentation rate (DAS28-ESR) is widely used as a measure of inflammatory disease activity in people with RA during clinical decision-making. In the UK, DAS28-ESR is used to determine eligibility for biologic therapies [1]. DAS28-ESR ≥3.2 can be used as a threshold for classifying active RA, and DAS28 is often used in clinical trials [2], or as a target for intensive treatment [3]. Current evidence supports these approaches as outcome measures in people with RA [4, 5].

Non-inflammatory mechanisms, through their effects on pain, can confound interpretation of DAS28-ESR ≥3.2 as a measure of active inflammation. Swollen joint count (SJC) and ESR are markers of inflammation. However, tender joint counts (TJCs) might be increased in people with centrally augmented pain, and the visual analogue scale for global health (VAS-GH) might be high in people fulfilling fibromyalgia (FM) classification [6, 7]. Persistent non-inflammatory pain can result in patients in remission being misclassified as having active inflammatory disease, leading to inappropriate clinical decisions to escalate therapy. Persistent non-inflammatory pain might compromise interpretation of outcomes in clinical trials. Furthermore, clinical tools that identify the subgroup of people with high DAS28 who also have high inflammation (rather than non-inflammatory pain) could help select people for inclusion in clinical trials or for anti-inflammatory treatment.

Several indices have been derived from DAS28-ESR components in an attempt to measure non-inflammatory contributions in people with RA. Pollard et al. have shown an association between tender minus swollen joint count (tender-swollen difference) and concurrent FM status or increased pain sensitivity [8]. We reported DAS28-P, defined as the proportion of DAS28-ESR attributable to patient-reported components (TJC and VAS-GH) [9]. DAS28-P was associated with pain severity, and predicted future pain in early RA [9], and in people commencing or changing biologic or non-biologic disease modifying treatments [10]. DAS28-P was also associated with increased pain sensitivity and concurrent FM in people with RA [7]. Evidence from one national registry suggested that the ratio of tender to swollen joint counts (tender:swollen ratio) might predict less good response of RA to biologic therapy [11], after being trichotomised into three groups. Each of these derived indices attempts to estimate the discordance between patient-reported symptoms and observed or laboratory measured inflammation. Other symptoms such as anxiety, depression and fatigue are strongly associated with chronic pain, and might reflect overlapping mechanisms within the central nervous system in people with RA [12]. We hypothesised that derived indices are associated with non-inflammatory pain and with patient-reported outcome measures of mental health and vitality or fatigue.

We sought to better understand DAS28-ESR, its components and the published derived indices in order to inform interpretation of non-inflammatory mechanisms in RA. To achieve this, we examined:- (1) The statistical properties of each index to show how each may be used in statistical analyses, including distribution (to show their continuum of values) and variability of repeat measurements (higher repeatability between clinicians improves the validity of a measure). (2) Associations of each index with pain and related patient-reported outcomes, and markers of central pain mechanisms, which are the primary objectives of their derivation. (3) Association with inflammatory disease activity (because indices of non-inflammatory mechanisms should not necessarily increase with increasing inflammation). (4) Prediction of future pain (non-inflammatory pain mechanisms might not be responsive to DMARD therapy and their presence might therefore predict poor pain prognosis).

## Methods

Datasets from four published studies were used to explore statistical characteristics of DAS28 components and derived indices of inflammatory or non-inflammatory disease activity; baseline data collected from participants in the British Society for Rheumatology Biologics Register (BSRBR) with active, established RA and a valid DAS28-ESR score who were (1) initiating anti-TNF therapy (BSRBR anti-TNF cohort; *n* = 10,813) [13], or (2) changing between non-biologic disease modifying anti-rheumatic drugs (DMARDs, BSRBR-Control cohort; *n* = 2992) [14], (3) participants in the Early Rheumatoid Arthritis Network (ERAN cohort; *n* = 813) [15], and (4) participants undergoing routine care in a hospital-based, cross sectional study exploring FM status and pain pressure thresholds (PPT group; *n* = 45) [7]. Written informed consent was obtained from all participants. Data were used only for participants with active disease, as defined by a DAS28-ESR ≥3.2. The PPT group included only complete case data available for all variables. Repeatability of the variables was determined from DAS28 assessments performed by multiple assessors in people with RA during training sessions completed during practice standardisation for clinicians in the ERAN study.

The BSRBR anti-TNF cohort is a national register, started in 2002 that tracked biologic naïve people with RA commencing their first anti-TNF agent. The BSRBR control cohort is a multicentre longitudinal study of people with RA who were changing to a new non-biologic treatment at recruitment, and was intended for use as a comparator to biologic cohorts. Both cohorts are coordinated by the University of Manchester. The BSRBR cohorts were approved by NHS North West Multicentre Research Ethics Committee (00/8/53). Baseline data were provided to the authors in de-identified, anonymous formats with no indication of the anti-TNF agent being used, nor the dates of enrolment and treatment. ERAN was a multicentre inception cohort study of newly diagnosed RA spanning the UK and Eire and commenced recruitment in 2002. The study was approved by Trent Research Ethics Committee (01/4/047). Baseline and follow up data up to 1 year were used in the current study. Participants in a hospital-based study of pain pressure thresholds and pain mechanisms in people with RA were recruited through routine annual outpatient appointments for RA management at Sherwood Forest Hospitals NHS Foundation Trust [7]. Ethical approval for this project was obtained from the East Midlands NREC – Nottingham 2 (13/EM/0047).

Participants with RA were eligible for inclusion if their DAS28-ESR ≥ 3.2.

Alterations and rearrangements of the DAS28 formula might possibly increase the variation in the derived scores compared to DAS28-ESR. Repeatability data were derived from DAS28-ESR assessments taken on the same day for each patient by each assessor across four clinical assessment training classes for rheumatology researchers. The total repeatability group contained 34 people with RA across the four sessions. People with RA were assessed in subgroups of 2–4, each individual assessed on two occasions separated by > 1 h by each of 3 to 7 rheumatology healthcare workers. Data were combined with single ESR measurements per individual to give DAS28-ESR or DAS28-P scores. Repeat measures of VAS-GH were available for a subset of 19 people with RA, while the remainder rated VAS-GH once.

The formulae for derived indices were:

$$ {\displaystyle \begin{array}{l}\mathrm{DAS}28\hbox{-} \mathrm{P}=\left({0.56}^{\ast}\surd \mathrm{TJC}+{0.014}^{\ast}\mathrm{VAS}-\mathrm{GH}\right)/\mathrm{DAS}28\hbox{-} \mathrm{ESR}\\ {}\mathrm{Tender}\hbox{-} \mathrm{swollen}\kern0.17em \mathrm{difference}=\mathrm{TJC}\hbox{--} \mathrm{SJC}\\ {}\mathrm{Tender}:\mathrm{swollen}\kern0.17em \mathrm{ratio}=\mathrm{TJC}/\mathrm{SJC}\end{array}} $$

Participants completed a range of self-report questionnaires addressing symptoms. Bodily Pain, Vitality and Mental Health were assessed in BSR-BR and ERAN groups using Short Form-36 (SF36) and norm-transformed values used for this study (SF36 subscales are negatively oriented, with lower scores reflecting worse quality of life). PPT participants completed the Beck Depression Inventory (BDI) [16]; State-Trait Anxiety Index short form (STAI-SF) [17]; Fatigue Severity Scale (FSS) [18]; Widespread Pain Index (WPI) [19]; Symptom Severity Scale (SSS) [20]. WPI and SSS questionnaires permitted FM classification according to American College of Rheumatology criteria 2010 [20].

PPT testing used an electronic pressure algometer with a laptop recording/display device and a patient switch (Somedic Sensebox). It was carried out by a single, trained observer (NJ) at the anterior tibia [7]. Low PPT (greater sensitivity) distant from affected joints is taken as an index of widespread (possibly central) sensitization.

CRP was included as a covariate marker of concurrent inflammatory disease activity that was not included in any of the derived indices. CRP was measured by clinical laboratories local to recruiting centres. Reported CRP values < 5 mg/ml were imputed with the value of 4 mg/ml. Ln-transformation of CRP was used to adjust for inflammation as a covariate and for regression analyses, because it displayed closer approximation to normality on Q-Q plots than did non-transformed CRP (data not shown).

### Statistical analysis

Repeatability was assessed by calculating the inter-observer measures of the same patient volunteer performed on the same day. The average coefficient of variation (CV; calculated as sd/mean) was calculated for each different measurement or index, as recommended as a measure of precision error [21], and is presented as a percentage. Differences between groups were assessed using T-tests and Cohen’s d effect size. Skewness and kurtosis were calculated for each variable [22]. Normality of distributions was assessed by Q-Q plots. For regression analyses, square root transformations of TJC and SJC; and natural logarithms were used for tender:swollen ratio and ESR.

Cross-sectional, baseline-only associations of DAS28-ESR, components or derived indices, transformed where necessary, with other outcomes were determined by linear regression of standardised variables providing B coefficients (95% CI) that could be compared between and across variables. Longitudinal analyses with Generalised Estimating Equations (GEE) used data from baseline to predict 3–6 month and 1 year SF36-Bodily Pain scores. The analyses were controlled for similar variables to other analyses of longitudinal pain performed in ERAN and BSRBR cohorts [9, 23], such as common disease markers for RA severity (disability, serology and disease duration), demographics or lifestyle (age, gender, BMI, smoking) and pain-related factors (mental health and fatigue). The effects of inflammation were adjusted for using CRP measures, as this was the available measure that was the most-independent compared to the other DAS28-ESR components. Analysis was performed using SPSS v22 (IBM, USA) and *p* < 0.05 was taken as statistical significance.

## Results

Demographics of each patient group are shown in Table 1. As expected, fewer participants satisfied 1987 ACR RA classification criteria in ERAN at baseline than in those other groups recruited from people with established RA. The BSRBR anti-TNF group displayed highest scores for DAS28 and its components.

**Table 1:** Demographics and clinical features of participant groups at baseline

	ERAN	BSRBR anti-TNF	BSRBR-Control	PPT study	Repeatability
*N*=	813	10,813	2992	45	34
%female	70%	76%	74%	77%	73%
Age (mean (sd))	57 (14)	56 (12)	60 (12)	60 (11)	60 (13)
Seropositive	59%	65%	58%	90%	79%
DAS28-ESR	5.2 (1.1)	6.6 (1.0)	5.4 (1.1)	4.7 (0.9)	4.8 (1.1)
ESR	34 (24)	46 (29)	37 (25)	21 (13)	39 (29)
SJC	7 (6)	11 (6)	6 (5)	1 (1)	5 (4)
TJC	9 (7)	16 (7)	9 (7)	11 (7)	6 (6)
VAS-GH	50 (24)	73 (20)	57 (23)	53 (23)	39 (20)
CRP	28 (37)	46 (42)	35 (40)	< 5	not collected

Eligible population: proportion (number) of cases from database with DAS28-ESR ≥ 3.2 and therefore included in this study. *TJC* tender joint count, *SJC* swollen joint count, *ESR* erythrocyte sedimentation rate, *VAS-GH* visual analogue scale-general health, *CRP* C-reactive protein (mg per dL)

### Measurement properties of DAS28-ESR, its components and derived indices

Tender:swollen ratio underwent logarithmic transformation to give a closer approximation to a normal distribution (which improves its measurement properties; Additional file 1: Figure S1 and Table S1). Transformed tender:swollen ratio data were therefore used in all analyses. Some measurements displayed prominent floor and ceiling effects. The proportions of cases that displayed either maximum or minimum scores were highest for tender:swollen ratio (≤15% minimum and ≤ 28% maximum depending upon participant group). SJCs were zero in 28% of PPT study, and 7% of ERAN participants, but ≤1% in BSRBR cohorts. Of BSRBR-anti-TNF cohort participants, 6% displayed SJC = 28, and 6% displayed VAS-GH = 100 mm. Other measures all showed ≤3% floor or ceiling prevalence. Inter-observer repeatability was indicated by coefficients of variation; TJC (41%), VAS-GH (16%), SJC (62%), DAS28-P (17%), tender-swollen difference (188%) and tender:swollen ratio (64%).

### Associations with pain and other patient-reported outcomes

DAS28-ESR and its components and each of the derived indices were significantly associated with Bodily Pain scores in each participant group (Additional file 1: Table S2). Cross-sectional associations with Bodily Pain scores were adjusted for CRP to explore possible dependence on non-inflammatory factors (Additional file 1: Table S3). Each study group showed multiple significant associations of worse Bodily Pain scores with DAS28-ESR, and also with the DAS28-ESR components TJC and VAS-GH. Bodily Pain scores were also significantly associated with DAS28-P; tender-swollen difference; and tender:swollen ratio. Less consistent findings were found for ESR and SJC.

Lower PPTs (more sensitive) at the anterior tibia were significantly associated with higher TJC, VAS-GH, DAS28-ESR, and DAS28-P. Lower PPTs were also significantly associated with tender-swollen difference, but not with SJC, ESR nor tender:swollen ratio (Table 2). DAS28-ESR, TJC, VAS-GH, DAS28-P and tender-swollen difference were higher in participants that satisfied criteria for FM, whereas SJC, ESR and tender:swollen ratio were not (Table 2).

**Table 2:** Associations of DAS28-related variables with pressure pain thresholds and fibromyalgia classification

Index	Medial Tibia PPT		Fibromyalgia (FM) classification				
	B (95% CI)	*p*	RA with FM	RA (no FM)	*p*	Difference	Effect size
TJC	**− 0.39 (− 0.63 to − 0.15)**	**0.002**	**14 (7)**	**8 (6)**	**0.01**	5.3	0.75
VAS-GH	**− 0.37 (− 0.61 to − 0.13)**	**0.003**	**67 (19)**	**42 (19)**	**< 0.001**	25	1.09
SJC	0.03 (− 0.23 to 0.28)	0.834	1 (1)	1 (2)	0.742	0.15	0.1
ESR	−0.10 (− 0.36 to 0.17)	0.464	23 (16)	20 (11)	0.51	2.7	0.2
DAS28	**− 0.53 (− 0.77 to − 0.29)**	**< 0.001**	**5.2 (0.8)**	**4.4 (0.8)**	**0.001**	0.79	0.93
DAS28-P	**−0.41 (− 0.62 to − 0.13)**	**0.003**	**0.56 (0.11)**	**0.48 (0.09)**	**0.008**	0.09	0.81
Tender-swollen difference	**−0.38 (− 0.62 to − 0.15)**	**0.002**	**12 (8)**	**7 (6)**	**0.011**	5.5	0.76
Tender:swollen ratio	**−0.48 (− 0.79 to − 0.18)**	**0.003**	8.4 (7.0)	6.2 (5.9)	0.363	2.1	0.33

Data from PPT study, *n* = 45. Linear regression between medal tibial pressure pain thresholds (PPT) and DAS28-related variables (lower values of PPTs indicate greater pain sensitivity). Means (sd); T-Tests and Cohens d effect size for differences between patients with RA ± fibromyalgia (FM). *TJC* tender joint count, *SJC* swollen joint count, *ESR* erythrocyte sedimentation rate, *VAS-GH* visual analogue scale-general health. DAS28-P = proportion of DAS28-ESR attributable to patient-reported components. No corrects were performed for multiple comparisons. Statistically significant findings highlighted in **bold**

Higher DAS28-ESR was associated with worse mental health, as measured by BDI (depression) or STAI-SF (anxiety), or SF36 Mental Health Score (distress; Table 3A). Higher DAS28-ESR was also associated with worse fatigue/lower vitality in each group (Table 3B). Associations with worse mental health or fatigue were stronger for VAS-GH and TJC than for SJC and ESR, and more consistently significant for DAS28-P and tender-swollen difference than for tender:swollen ratio (Table 3A, B).

**Table 3:** Associations of DAS28-related variables with mental health and vitality or fatigue

A										
	BSRBR antiTNF		BSRBR-Control		ERAN		PPT study			
	SF36-Mental Health Score		SF36-Mental Health Score		SF36-Mental Health Score		Beck Depression Index		State Trait Anxiety Index	
	B (95% CI)	*p*	B (95% CI)	*p*	B (95% CI)	*p*	B (95% CI)	*p*	B (95% CI)	*p*
TJC	**− 0.12 (− 0.14 to − 0.10)**	**< 0.001**	**− 0.17 (− 0.21 to − 0.13)**	**< 0.001**	**− 0.19 (− 0.26 to − 0.11)**	**< 0.001**	**0.29 (0.01 to 0.57)**	**0.044**	**0.36 (0.08 to 0.63)**	**0.012**
VAS-GH	**− 0.19 (− 0.21 to − 0.17)**	**< 0.001**	**− 0.28 (− 0.32 to − 0.24)**	**< 0.001**	**− 0.36 (− 0.43 to − 0.29)**	**< 0.001**	**0.47 (0.21 to 0.73)**	**0.001**	**0.45 (0.19 to 0.70)**	**0.001**
SJC	**−0.04 (− 0.06 to − 0.02)**	**< 0.001**	**−0.05 (− 0.09 to − 0.01)**	**0.017**	−0.01 (− 0.09 to 0.07)	0.735	−0.19 (− 0.48 to 0.09)	0.184	−0.22 (− 0.50 to 0.06)	0.125
ESR	**−0.07 (− 0.09 to − 0.05)**	**< 0.001**	−0.04 (− 0.08 to 0.00)	0.078	**−0.11 (− 0.18 to − 0.03)**	**0.006**	0.15 (− 0.16 to 0.46)	0.338	0.01 (− 0.30 to 0.31)	0.967
DAS28	**−0.17 (− 0.19 to − 0.15)**	**< 0.001**	**−0.21 (− 0.27 to − 0.17)**	**< 0.001**	**−0.25 (− 0.32 to − 0.18)**	**< 0.001**	**0.33 (0.02 to 0.64)**	**0.039**	**0.33 (0.03 to 0.63)**	**0.034**
DAS28-P	**−0.10 (− 0.12 to − 0.08)**	**< 0.001**	**−0.18 (− 0.22 to − 0.14)**	**< 0.001**	**−0.23 (− 0.30 to − 0.15)**	**< 0.001**	**0.37 (0.09 to 0.65)**	**0.012**	**0.41 (0.13 to 0.69)**	**0.005**
Tender-swollen difference	**−0.09 (− 0.11 to − 0.07)**	**< 0.001**	**−0.14 (− 0.18 to − 0.10)**	**< 0.001**	**−0.18 (− 0.25 to − 0.10)**	**< 0.001**	**0.31 (0.04 to 0.59)**	**0.027**	**0.39 (0.12 to 0.66)**	**0.006**
Tender:swollen ratio	**−0.06 (− 0.08 to − 0.04)**	**< 0.001**	**−0.10 (− 0.14 to − 0.06)**	**< 0.001**	−0.11 (− 0.22 to 0.01)	0.060	0.27 (− 0.08 to 0.61)	0.128	0.24 (− 0.08 to 0.57)	0.135
B										
	BSRBR anti-TNF		BSRBR control		ERAN		PPT study			
	SF36-Vitality		SF36-Vitality		SF36-Vitality		Fatigue Severity Scale			
	B (95% CI)	*p*	B (95% CI)	*p*	B (95% CI)	*p*	B (95% CI)	*p*		
TJC	**−0.13 (− 0.15 to − 0.11)**	**< 0.001**	**− 0.17 (− 0.21 to − 0.13)**	**< 0.001**	**− 0.27 (− 0.34 to − 0.20)**	**< 0.001**	**0.30 (0.01 to 0.58)**	**0.042**		
VAS-GH	**− 0.19 (− 0.21 to − 0.17)**	**< 0.001**	**− 0.26 (− 0.30 to − 0.22)**	**< 0.001**	**−0.36 (− 0.43 to − 0.29)**	**< 0.001**	**0.57 (0.32 to 0.81)**	**< 0.001**		
SJC	**−0.05 (− 0.07 to − 0.03)**	**< 0.001**	**−0.06 (− 0.10 to − 0.02)**	**0.002**	−0.07 (− 0.14 to 0.01)	0.100	−0.11 (− 0.41 to 0.19)	0.473		
ESR	**−0.06 (− 0.08 to − 0.04)**	**< 0.001**	**−0.09 (− 0.13 to − 0.05)**	**0.002**	**−0.14 (− 0.22 to − 0.07)**	**< 0.001**	0.16 (− 0.15 to 0.47)	0.304		
DAS28	**− 0.18 (− 0.20 to − 0.15)**	**< 0.001**	**−0.25 (− 0.29 to − 0.21)**	**< 0.001**	**−0.33 (− 0.40 to − 0.26)**	**< 0.001**	**0.43 (0.12 to 0.73)**	**0.007**		
DAS28-P	**−0.11 (− 0.13 to − 0.09)**	**< 0.001**	**−0.17 (− 0.21 to − 0.13)**	**< 0.001**	**−0.28 (− 0.35 to − 0.21)**	**< 0.001**	**0.38 (0.08 to 0.68)**	**0.013**		
Tender-swollen difference	**−0.09 (− 0.11 to − 0.07)**	**< 0.001**	**−0.13 (− 0.17 to − 0.09)**	**< 0.001**	**− 0.22 (− 0.29 to − 0.15)**	**< 0.001**	**0.31 (0.03 to 0.60)**	**0.033**		
Tender:swollen ratio	**−0.07 (− 0.09 to − 0.05)**	**< 0.001**	**−0.09 (− 0.13 to − 0.05)**	**< 0.001**	**−0.17 (− 0.27 to − 0.08)**	**0.001**	0.34 (− 0.05 to 0.74)	0.087		

A: Mental health scores and B: Fatigue scores, with their associations with DAS28-related variables. Standardised linear regression coefficients, 95% confidence intervals and *p* values. *TJC* tender joint count, *SJC* swollen joint count, *ESR* erythrocyte sedimentation rate, *VAS-GH* visual analogue scale-general health. DAS28-P = proportion of DAS28-ESR attributable to patient-reported components. No corrects were performed for multiple comparisons. Statistically significant findings highlighted in **bold**

### Associations between DAS28-related variables, DAS28-ESR and CRP

Indices of non-inflammatory pain might be expected to be independent of inflammation levels. Each individual DAS28-ESR component and derived index displayed the expected significant positive associations with DAS28-ESR in each participant group (Table 4). Associations of each derived index with DAS28-ESR appeared to have smaller coefficients than those of the individual DAS28-ESR components (Table 4). The weakest association of DAS28-ESR was with tender:swollen ratio. The expected positive associations were found between DAS28-ESR components and serum CRP level. Conversely, none of the derived indices displayed significant positive correlation with serum CRP (Table 4). DAS28-P was significantly negatively associated with serum CRP level in all groups, whereas negative associations with tender-swollen differences and tender:swollen ratios were weaker than with DAS28-P (Table 4).

**Table 4:** Associations of DAS28-related variables with DAS28-ESR and CRP

		BSRBR anti-TNF		BSRBR-Control		ERAN	
		B (95% CI)	*p*	B (95% CI)	*p*	B (95% CI)	*p*
DAS28	TJC	**0.72 (0.71 to 0.73)**	**< 0.001**	**0.76 (0.74 to 0.78)**	**< 0.001**	**0.59 (0.55 to 0.62)**	**< 0.001**
	VAS-GH	**0.49 (0.47 to 0.50)**	**< 0.001**	**0.62 (0.59 to 0.65)**	**< 0.001**	**0.46 (0.42 to 0.51)**	**< 0.001**
	SJC	**0.56 (0.55 to 0.58)**	**< 0.001**	**0.60 (0.58 to 0.62)**	**< 0.001**	**0.48 (0.45 to 0.52)**	**< 0.001**
	ESR	**0.49 (0.48 to 0.51)**	**< 0.001**	**0.53 (0.51 to 0.56)**	**< 0.001**	**0.41 (0.37 to 0.45)**	**< 0.001**
	DAS28	Not applicable					
	DAS28-P	**0.19 (0.17 to 0.20)**	**< 0.001**	**0.29 (0.26 to 0.32)**	**< 0.001**	**0.24 (0.19 to 0.29)**	**< 0.001**
	Tender-swollen difference	**0.25 (0.24 to 0.27)**	**< 0.001**	**0.36 (0.32 to 0.39)**	**< 0.001**	**0.15 (0.11 to 0.20)**	**< 0.001**
	Tender:swollen ratio	**0.11 (0.09 to 0.12)**	**< 0.001**	**0.13 (0.10 to 0.16)**	**< 0.001**	**0.07 (0.02 to 0.12)**	**0.011**
CRP	TJC	−0.02 (− 0.05 to 0.01)	0.161	**0.09 (0.02 to 0.16)**	**0.010**	**0.17 (0.07 to 0.26)**	**< 0.001**
	VAS-GH	**0.17 (0.14 to 0.20)**	**< 0.001**	**0.21 (0.13 to 0.28)**	**< 0.001**	**0.17 (0.08 to 0.26)**	**< 0.001**
	SJC	**0.10 (0.07 to 0.13)**	**< 0.001**	**0.11 (0.05 to 0.17)**	**0.001**	**0.23 (0.13 to 0.32)**	**< 0.001**
	ESR	**0.58 (0.55 to 0.60)**	**< 0.001**	**0.56 (0.51 to 0.62)**	**< 0.001**	**0.62 (0.55 to 0.69)**	**< 0.001**
	DAS28	**0.36 (0.33 to 0.39)**	**< 0.001**	**0.47 (0.40 to 0.55)**	**< 0.001**	**0.45 (0.37 to 0.53)**	**< 0.001**
	DAS28-P	**−0.31 (−0.34 to − 0.28)**	**< 0.001**	**− 0.28 (− 0.36 to − 0.20)**	**< 0.001**	**−0.20 (− 0.30 to − 0.11)**	**< 0.001**
	Tender-swollen difference	**− 0.10 (− 0.13 to − 0.07)**	**< 0.001**	−0.01 (− 0.08 to 0.06)	0.851	−0.03 (− 0.13 to 0.06)	0.487
	Tender:swollen ratio	**−0.11 (− 0.14 to − 0.08)**	**< 0.001**	**−0.08 (− 0.15 to − 0.01)**	**0.020**	−0.08 (− 0.18 to 0.02)	0.104

Linear regression of z-transformed variables. Regression coefficients (95% CI) and *p* values for associations with the inflammatory measures, DAS28 and CRP. CRP data were ln-transformed prior to regression analysis. *TJC* tender joint count, *SJC* swollen joint count, *ESR* erythrocyte sedimentation rate, *VAS-GH* visual analogue scale-general health. DAS28-P = proportion of DAS28-ESR attributable to patient-reported components. No corrects were performed for multiple comparisons. Statistically significant findings highlighted in **bold**

### Longitudinal prediction of pain

The longitudinal prediction of pain was tested for each of the DAS28-ESR components and derived indices using GEE analyses. Baseline DAS28-ESR, derived indices and DAS28-ESR components each predicted worse pain prognosis up to 1 year in univariate analyses (Table 5). Baseline tender-swollen difference and tender:swollen ratio were significantly associated with worse pain prognosis in all longitudinal cohorts using multivariable GEE analysis adjusting for baseline pain, inflammation measured by CRP, and other potential confounders (Table 5). High DAS28-P was significantly associated with worse pain prognosis in ERAN and BSRBR non-biologic cohorts, but not in the BSRBR anti-TNF cohort after multivariable analysis (Table 5). Associations of DAS28-ESR, SJC, TJC or VAS-GH with pain prognosis was significant in only one of the three cohorts for each (Table 5).

**Table 5:** Longitudinal associations of baseline DAS28-related variables with pain at follow up

	ERAN				BSRBR anti-TNF				BSRBR-Non-Biologic			
	Univariate		Multivariable		Univariate		Multivariable		Univariate		Multivariable	
	B (95% CI)	*p*	B (95% CI)	*p*	B (95% CI)	*p*	B (95% CI)	*p*	B (95% CI)	*p*	B (95% CI)	*p*
TJC	**−0.28 (− 0.34 to − 0.22)**	**< 0.001**	−0.09 (− 0.21 to 0.03)	0.131	**−0.10 (− 0.12 to − 0.08)**	**< 0.001**	−0.02 (− 0.05 to 0.01)	0.264	**−0.23 (− 0.26 to − 0.19)**	**< 0.001**	**−0.10 (− 0.16 to − 0.05)**	**< 0.001**
SJC	**−0.09 (− 0.15 to − 0.03)**	**0.004**	0.07 (− 0.01 to 0.16)	0.087	−0.02 (− 0.04 to 0.00)	0.090	**0.03 (0.00 to 0.06)**	**0.029**	**−0.04 (− 0.08 to − 0.01)**	**0.019**	−0.02 (− 0.07 to 0.03)	0.523
VAS-GH	**−0.36 (− 0.44 to − 0.29)**	**< 0.001**	−0.10 (− 0.23 to 0.02)	0.102	**−0.14 (− 0.16 to − 0.12)**	**< 0.001**	−0.02 (− 0.05 to 0.01)	0.139	**−0.29 (− 0.32 to − 0.26)**	**< 0.001**	**−0.14 (− 0.20 to − 0.09)**	**< 0.001**
ESR	**−0.18 (− 0.25 to − 0.11)**	**< 0.001**	0.07 (− 0.07 to 0.20)	0.338	**−0.07 (− 0.09 to − 0.05)**	**< 0.001**	0.01 (− 0.02 to 0.03)	0.695	**−0.09 (− 0.13 to − 0.05)**	**< 0.001**	−0.04 (− 0.11 to 0.04)	0.349
DAS28	**−0.31 (− 0.38 to − 0.23)**	**< 0.001**	−0.04 (− 0.17 to 0.09)	0.564	**−0.14 (− 0.16 to − 0.12)**	**< 0.001**	−0.02 (− 0.04 to 0.01)	0.176	**−0.25 (− 0.28 to − 0.21)**	**< 0.001**	**−0.13 (− 0.19 to − 0.07)**	**< 0.001**
DAS28-P	**−0.26 (− 0.34 to − 0.19)**	**< 0.001**	**−0.15 (− 0.27 to − 0.04)**	**0.007**	**−0.07 (− 0.09 to − 0.05)**	**< 0.001**	−0.02 (− 0.04 to 0.00)	0.089	**−0.20 (− 0.24 to − 0.17)**	**< 0.001**	**−0.14 (− 0.23 to − 0.05)**	**0.002**
Tender-swollen difference	**−0.25 (− 0.32 to − 0.18)**	**< 0.001**	**−0.14 (− 0.24 to − 0.04)**	**0.004**	**−0.08 (− 0.10 to − 0.06)**	**< 0.001**	**−0.03 (− 0.05 to − 0.00)**	**0.019**	**−0.20 (− 0.23 to − 0.16)**	**< 0.001**	**−0.11 (− 0.17 to − 0.05)**	**0.001**
Tender:swollen ratio	**−0.17 (− 0.23 to − 0.10)**	**< 0.001**	**−0.14 (− 0.21 to 0.06)**	**< 0.001**	**−0.06 (− 0.08 to − 0.05)**	**< 0.001**	**−0.03 (− 0.05 to − 0.01)**	**0.007**	**−0.15 (− 0.19 to − 0.11)**	**< 0.001**	**−0.08 (− 0.14 to − 0.02)**	**0.016**

Generalised Estimating Equations for prediction of longitudinal pain (3/6 month and 1 year SF36-Bodily Pain). Univariate analyses show the associations between baseline DAS28-related variables and longitudinal pain with no adjustments. Multivariable analyses were similar, but with adjustments for baseline measures of pain, DAS28, CRP, seropositive, age, gender, BMI, smoking, SF36-physical function, SF36-vitality, SF36-mental health. *TJC* tender joint count, *SJC* swollen joint count, *ESR* erythrocyte sedimentation rate, *VAS*-GH visual analogue scale-general health. DAS28-P = proportion of DAS28-ESR attributable to patient-reported components. No corrects were performed for multiple comparisons. Statistically significant findings highlighted in **bold**

## Discussion

We have shown that indices derived from DAS28-ESR components can give insight into the non-inflammatory contributions to apparent inflammatory disease activity in people with RA. Tender-swollen difference might have some advantages over other derived indices, although head-to-head statistical comparisons were not made. Derived indices were consistent with being measures of non-inflammatory factors, as they were less strongly associated with DAS28-ESR than were individual DAS28-ESR components; and tender-swollen difference was not consistently associated with CRP. Tender-swollen difference and DAS28-P each was positively associated with FM status, and with PPT evidence of neuronal sensitisation, as found previously [8, 24]. They displayed normal-like distributions and were without notable floor or ceiling effects. Tender-swollen difference more consistently predicted pain outcomes than did DAS28-P. DAS28-derived indices have potential as research tools, to help interpret DAS28-ESR as a measure of inflammatory disease activity, and to help predict those who might benefit from additional interventions aiming to reduce the long-term burden of pain in people with RA.

DAS28-ESR is an important measure of inflammatory disease activity in RA [25–29]. Our analyses confirm a strong association between DAS28-ESR or its components and self-reported pain, as would be expected where inflammation drives RA symptoms. However, VAS-GH and TJC, and therefore DAS28-ESR, are strongly influenced by non-inflammatory pain mechanisms [7]. Some authors have found benefits from analysing SJC + ESR combined and contrasting to TJC + VAS-GH combined. This may be a way to further examine people in clinical remission with low inflammation, but high pain [30]. Furthermore, as DAS28 shares many components with the Clinical Disease Activity Index [31] and the Simplified Disease Activity Index [32], plus some of the criteria for Boolean Remission [33], there may be scope for cross-validation between different indices.

We have shown that indices derived from DAS28-ESR components are associated with pain, while displaying little dependence on inflammation, as measured by CRP. In cross-sectional analyses, DAS28-ESR and the derived indices were similarly associated with pain and other symptoms. However, each of the derived indices predicted 1 year pain prognosis more strongly than did DAS28-ESR or each of its components. However, examining the exact mechanisms for pain prognosis is a complex task. Modern treatment, including biologic therapies, might counteract the importance of synovitis for pain prognosis. Pain might be more likely to persist where non-inflammatory mechanism make an important contribution and therefore pain outcomes might be best predicted by derived indices that reflect those non-inflammatory mechanisms.

Our data extend previous findings that DAS28-P predicted pain following initiation of biologic or non-biologic DMARDS [9], and that swollen:tender ratio predicted response to biologic treatment [11]. We demonstrate prediction of pain outcomes also by tender-swollen difference, and that pain prediction by derived indices persists after adjusting for other baseline demographic and clinical factors known to influence pain, including measures of inflammatory disease activity, psychological distress, obesity and smoking status.

We found that pain prediction by baseline values of derived indices were weaker in the BSRBR anti-TNF cohort than in the other two studies, consistent with our previous analyses [9, 10]. It is possible that TNF blockade inhibits non-inflammatory as well as inflammatory pain mechanisms [34], or that more effective suppression of an inflammatory drive to central sensitisation might also inhibit non-inflammatory pain mechanisms [35]. Confounding by the higher baseline RA severity in the BSRBR biologics cohort might also contribute to differences in pain prediction. DAS28-P did not predict EULAR-defined inflammatory disease responses 3 months after initiation of biologic therapy in another study [36], and factors that predict pain response to treatment might differ from those predicting inflammatory disease suppression.

Low PPTs indicate neuronal sensitisation and, when observed at a distance from affected joints, might reflect central sensitisation [37]. Concurrent classification of FM is common in people with RA [7] and is considered also to reflect central sensitisation. Derived indices displayed significant associations with PPTs and FM classification, suggesting that they might reflect central sensitisation in RA. Central sensitisation might indicate comorbid conditions such as FM or result from sustained nociceptive input from joint inflammation [12] or other co-morbidities, such as osteoarthritis and low back pain. We did not observe significant associations between SJC or ESR and PPTs or FM classification, indicating that ongoing inflammation might not be necessary to sustain sensitisation in RA, although inflammation might yet contribute to the development of sensitisation in earlier disease.

Self-reported fatigue and poor mental health scores are associated with worse pain and greater central sensitisation in people with RA [38, 39]. DAS28-ESR was significantly associated with fatigue and mental health in each study group. However, SJC and ESR were less consistently or less strongly (smaller coefficients) associated with fatigue or mental health than were TJC and VAS-GH, paralleling the various associations seen with bodily pain. Furthermore, we now extend our previous findings on pain [7] to show that derived indices were significantly associated with fatigue and mental health scores in both early and established disease. Fatigue and/or poor mental health might, therefore, be additional centrally mediated non-inflammatory mechanisms that confound inflammatory disease assessment using DAS28-ESR.

Derived indices displayed less positive association with CRP than did DAS28-ESR or its components, and therefore have some external validity as measures of non-inflammatory mechanisms. Testing against additional measures of inflammation, for example using ultrasound or magnetic resonance imaging, might further extend these findings. Tender-swollen difference displayed some advantages over the other derived measures, in being simple to calculate, displaying a normal distribution without important floor and ceiling effects, displaying consistent associations with pain, PPT and FM status, and consistently predicting worse pain outcome across all three cohort studies. The inclusion of four items in DAS28-P might improve its validity over indices derived from only two items, but the significant negative association of DAS28-P with CRP, possibly due to the inclusion of ESR in its denominator, suggests potential confounding by inflammation. DAS28-P displayed a lower coefficient of variation than did the tender-swollen difference. However, the coefficient of variation statistic tends towards infinity where mean values approach zero, and this might underestimate the repeatability of the tender-swollen difference. It showed similar standardised coefficients and confidence intervals with other indices, and we believe that it has similar validity to DAS28-P when used to analyse these data. Inclusion of a single ESR measurement might also have led us to overestimate the repeatability of DAS28-P. Tender:swollen ratio had a number of disadvantages including floor and ceiling effects, and skewness that necessitated transformation prior to parametric analysis. When the swollen:tender ratio was derived, it was split into three response levels to deal with the effects of zero values [11], whereas this analysis focussed on comparisons of continuous indices. Tender-swollen difference might be a preferred continuous derived index for studying non-inflammatory contributions to RA pain.

Our study is subject to several limitations, despite the availability of data from several large groups with early or established RA. The consistent pattern of findings for each index is presented across a variety of data sources. As most of the comparisons were complementary, no corrections were made for multiple testing. Derived indices have value in epidemiology or secondary data analyses where more specific measures of non-inflammatory mechanisms such as central sensitisation are unfeasible or not available. However, their interpretation as surrogates for pain sensitivity or central sensitisation should be cautious in the context of other potential confounders such as mental health or fatigue. In the absence of a gold-standard measure of central sensitisation or non-inflammatory pain, we have used PPTs and bodily pain as surrogates. Using other surrogate measures might have altered the study findings. Further research is required to determine which specific non-inflammatory disease mechanisms modulate assessment of inflammation using DAS28-ESR. Our study used CRP as a measure of inflammation, although some patients with RA might have continuing inflammatory disease activity despite normal CRP, in particular since the introduction of interleukin-6 blockade. CRP was selected because it was not included in any of the DAS28-related variables, and so would be the least likely to be a confounder. Other measures of synovitis might reveal possible associations of derived indices with residual synovitis. Lack of formal reliability data is a limitation, and necessitated use of CV values. The repeatability data are limited by their collection from a large number of practitioners with varying experience, and may reflect the “real world” variability, rather than an optimal variability. CV values of VAS-GH might be underestimated because it was not possible to blind participants to previous instructions or responses during repeated assessment by successive practitioners. It should be noted that other definitions of disease activity, treatment response and remission are available [40–42]. Data from observational cohort studies cannot distinguish effects on treatment from those on placebo response or natural history. Future studies should therefore also test the ability of derived measures to predict response to treatments within the context of randomised controlled trials, either targeting synovitis or non-inflammatory disease mechanisms.

Non-inflammatory contributions are of particular clinical importance where DAS28-ESR suggests active inflammatory disease. Overestimation of synovitis might result in inappropriate exposure of patients to potentially toxic or expensive treatments, and underestimation of inflammatory disease suppression might lead to discontinuation of treatments that would otherwise prevent joint damage and subsequent disability. The presence of non-inflammatory pain mechanisms does not, however, exclude concurrent inflammation, and even moderate disease activity is longitudinally associated with both poor function and joint damage [28]. Specific measures of synovitis such as ultrasound assessment might help guide treatment titration to achieve low disease activity or remission in all cases, particularly where non-inflammatory mechanisms might conceal contributions of persistent synovitis to DAS28-ESR. Non-inflammatory mechanisms underlying important symptoms such as pain, fatigue or impaired mental health are important even where DAS28-ESR suggests that inflammation is well controlled. Derived measures are unlikely to be appropriate in people with remission of inflammatory disease, where more experimental measures such as PPT or other forms of quantitative sensory testing might reveal non-inflammatory pain mechanisms. Where DAS28-ESR < 3.1, DAS28-P displays a skewed distribution and high measurement errors are generated by ratios in which denominators are close to zero [9].

## Conclusion

In conclusion, we demonstrate the potential of derived indices as measures of non-inflammatory mechanisms in people with apparently active RA (DAS28-ESR ≥ 3.2). Our analyses suggest that tender-swollen difference, DAS28-P and tender:swollen ratio are surrogate indices of non-inflammatory pain mechanisms, and we propose that central sensitisation is a likely candidate. DAS28-ESR remains a valuable measure of active synovitis, which continues to facilitate the development of disease modifying treatments and helps target treatments those to those who gain most benefit [4, 5, 43, 44]. Derived indices, such as tender-swollen difference, conveniently assist interpretation of DAS28-ESR as a measure of inflammatory disease activity. With further research to establish thresholds, they may have potential to help identify people with RA who might benefit from interventions that target non-inflammatory mechanisms in order to improve their pain prognosis.

## Additional file

Additional file 1: **Figure S1.** Measurement properties of each index. **Table S1.** Measurement properties of DAS28-related variables. **Table S2.** Cross-sectional associations between DAS28-related variables and pain at baseline assessment. **Table S3.** Cross-sectional associations between DAS28-related variables and pain after adjustment for CRP. (DOCX 139 kb)

## Abbreviations

BDI: Beck Depression Inventory
BSRBR: British Society for Rheumatology Biologics Register
CRP: C-reactive protein
DAS28: Disease activity score for 28 joints
ERAN: Early Rheumatoid Arthritis Network
ESR: Erythrocyte sedimentation rate
FM: Fibromyalgia
FSS: Fatigue Severity Scale
PPT: Pressure pain thresholds
SJC: Swollen joint count
SSS: Symptom Severity Scale
STAI-SF: State-Trait Anxiety Index short form
TJC: Tender joint count
VAS-GH: Visual analogue scale
WPI: Widespread Pain Index
