# Supplementary material for: Interpretation of DAS28 and its components in the assessment of inflammatory and non-inflammatory aspects of rheumatoid arthritis
Source: BMC Rheumatol. 2018 Mar 23;2:8. doi: 10.1186/s41927-018-0016-9 (PMC6390559; doi:10.1186/s41927-018-0016-9)
Supplement: Supplementary file 1 — Figure S1. Measurement properties of each index. Table S1. Measurement properties of DAS28-related variables. Table S2. Cross-sectional associations between DAS28-related variables and pain at baseline assessment. Table S3. Cross-sectional associations between DAS28-related variables and pain after adjustment for CRP. (DOCX 139 kb) [file 41927_2018_16_MOESM1_ESM.docx]

**Additional file 1**

**Table S1: Measurement properties of DAS28-related variables**

|  | BSRBR anti-TNF | |  | BSRBR controls | |  | ERAN | |  | PPT in RA | |
| --- | --- | --- | --- | --- | --- | --- | --- | --- | --- | --- | --- |
|  | Kurtosis (se) | Skewness (se) |  | Kurtosis (se) | Skewness (se) |  | Kurtosis (se) | Skewness (se) |  | Kurtosis (se) | Skewness (se) |
| TJC | -0.99(0.045) | -0.01 (0.023) |  | 0.24(0.085) | 0.93(0.043) |  | 0.30(0.17) | 0.96(0.08) |  | -0.19(0.68) | 0.83(0.35) |
| VAS | 0.99 (0.046) | -0.97(0.023) |  | -0.58(0.085) | -0.24(0.043) |  | -0.77(0.17) | -0.05(0.08)- |  | -1.20(0.68) | -0.13(0.35) |
| SJC | -0.35(0.045) | 0.46(0.023) |  | 2.11(0.085) | 1.36(0.043) |  | 0.97(0.17) | 1.11(0.08) |  | 1.84(0.68) | 1.33(0.35) |
| ESR | 0.40 (0.047) | 0.81(0.023) |  | 1.01(0.089) | 1.10(0.045) |  | 1.08(0.17) | 1.19(0.08) |  | 1.60(0.68) | 1.19(0.35) |
| DAS28 | 0.66 (0.047) | -0.36(0.023) |  | -0.37(0.089) | 0.31(0.045) |  | -0.37(0.17) | 0.37(0.08) |  | -0.52(0.68) | 0.10(0.68) |
| DAS28-P | 1.82 (0.047) | -0.30(0.023) |  | 1.36(0.090) | -0.53(0.045) |  | 1.2(0.17) | -0.48(0.08) |  | 0.09(0.68) | 0.09(0.35) |
| TJC-SJC difference | 0.66(0.045) | 0.01(0.023) |  | 2.10(0.085) | 0.38(0.043) |  | 1.96(0.17) | 0.24(0.08) |  | 0.07(0.68) | 0.88(0.35) |
| TJC/SJC ratio | 48.65(0.046) | 5.46(0.023) |  | 30.3(0.088) | 4.2(0.044) |  | 21.6(0.17) | 4.00(0.08) |  | 3.4(0.80) | 1.8(0.41) |
| LN(TJC/SJC ratio) | 2.35(0.46) | -0.31(0.023) |  | 1.29(0.085) | -0.18 (0.045) |  | 1.32(0.17) | -0.03(0.08) |  | -0.29(0.80) | --0.28(0.41) |

Kurtosis and skewness (standard error) for each variable are shown. DAS28-P=proportion of DAS28-ESR attributable to patient-reported components; TJC=tender joint count, SJC=swollen joint count, ESR=erythrocyte sedimentation rate, VAS=visual analogue scale-general health.

**Table S2: Cross-sectional associations between DAS28-related variables and pain at baseline assessment**

|  | BSRBR anti-TNF | |  | BSRBR-Control | |  | ERAN | |  | PPT | |
| --- | --- | --- | --- | --- | --- | --- | --- | --- | --- | --- | --- |
| Index | SF36-Bodily Pain Score | |  | SF36-Bodily Pain Score | |  | SF36-Bodily Pain Score | |  | Medial Tibia PPT | |
|  | B (95% CI) | p |  | B (95% CI) | p |  | B (95% CI) | p |  | B (95% CI) | p |
| TJC | **-0.18 (-0.20 to -0.16)** | **<0.001** |  | **-0.25 (-0.29 to -0.21)** | **<0.001** |  | **-0.34 (-0.41 to -0.27)** | **<0.001** |  | **-0.49 (-0.74 to -0.23)** | **<0.001** |
| VAS | **-0.33 (-0.35 to -0.31)** | **<0.001** |  | **-0.38 (-0.42 to -0.34)** | **<0.001** |  | **-0.52 (-0.58 to -0.45)** | **<0.001** |  | **-0.46 (-0.72 to -0.21)** | **0.001** |
| SJC | **-0.07 (-0.09 to -0.05)** | **<0.001** |  | **-0.11 (-0.15 to -0.07)** | **<0.001** |  | **-0.16 (-0.24 to -0.09)** | **<0.001** |  | 0.03 (-0.26 to 0.31) | 0.859 |
| ESR | **-0.14 (-0.16 to -0.13)** | **<0.001** |  | **-0.11 (-0.15 to -0.08)** | **<0.001** |  | **-0.17 (-0.24 to -0.09)** | **<0.001** |  | -0.18 (-0.46 to 0.11) | 0.227 |
| DAS28 | **-0.30 (-0.32 to -0.28)** | **<0.001** |  | **-0.37 (-0.42 to -0.33)** | **<0.001** |  | **-0.46 (-0.52 to -0.39)** | **<0.001** |  | **-0.61 (-0.84 to -0.38)** | **<0.001** |
| DAS28-P | **-0.14 (-0.16 to -0.12)** | **<0.001** |  | **-0.23 (-0.27 to -0.19)** | **<0.001** |  | **-0.35 (-0.42 to -0.28)** | **<0.001** |  | **-0.46 (-0.72 to -0.20)** | **0.001** |
| Tender-swollen difference | **-0.13 (-0.14 to -0.11)** | **<0.001** |  | **-0.18 (-0.21 to -0.14)** | **<0.001** |  | **-0.21 (-0.28 to -0.14)** | **<0.001** |  | **-0.48 (-0.73 to -0.22)** | **<0.001** |
| Tender:swollen ratio | **-0.09 (-0.11 to -0.07)** | **<0.001** |  | **-0.24 (-0.30 to --0.17)** | **<0.001** |  | **-0.16 (-0.22 to -0.11)** | **<0.001** |  | **-0.50 (-0.81 to -0.19)** | **0.002** |

Linear regression of z-transformed variables. Regression coefficients (95% CI) and p values for associations with pain measures. Negative B values represent variables associating with worse pain, because SF36-Bodily Pain is a negative index and lower pressure pain detection thresholds (PPT) indicate greater sensitivity. TJC=tender joint count, SJC=swollen joint count, ESR=erythrocyte sedimentation rate, VAS=visual analogue scale-general health. DAS28-P=proportion of DAS28-ESR attributable to patient-reported components. No corrects were performed for multiple comparisons. Statistically significant findings highlighted in **bold**.

**Table S3: Cross-sectional associations between DAS28-related variables and pain after adjustment for CRP**

|  | BSRBR anti-TNF | |  | BSRBR-Control | |  | ERAN | |  | PPT | |
| --- | --- | --- | --- | --- | --- | --- | --- | --- | --- | --- | --- |
| Index | SF36-Bodily Pain Score | |  | SF36-Bodily Pain Score | |  | SF36-Bodily Pain Score | |  | Medial Tibia PPT | |
|  | B (95% CI) | p |  | B (95% CI) | p |  | B (95% CI) | p |  | B (95% CI) | p |
| TJC | **-0.19 (-0.23 to -0.16)** | **<0.001** |  | **-0.18 (-0.25 to -0.10)** | **<0.001** |  | **-0.34 (-0.43 to -0.24)** | **<0.001** |  | **-0.41 (-0.64 to -0.17)** | **0.001** |
| VAS | **-0.32 (-0.35 to -0.29)** | **<0.001** |  | **-0.28 (-0.36 to -0.19)** | **<0.001** |  | **-0.47 (-0.55 to -0.39)** | **<0.001** |  | **-0.43 (0.61 to -0.12)** | **0.004** |
| SJC | **-0.06 (-0.09 to -0.03)** | **<0.001** |  | -0.05 (-0.11 to 0.02) | 0.170 |  | **-0.15 (-0.25 to -0.05)** | **0.003** |  | 0.02 (-0.24 to 0.28) | 0.874 |
| ESR | **-0.08 (-0.12 to -0.04)** | **<0.001** |  | -0.05 (-0.14 to 0.05) | 0.322 |  | -0.06 (-0.18 to 0.07) | 0.354 |  | -0.09 (-0.44 to 0.28) | 0.656 |
| DAS28 | **-0.28 (-0.32 to -0.25)** | **<0.001** |  | **-0.25 (-0.35 to -0.16)** | **<0.001** |  | **-0.44 (-0.53 to -0.34)** | **<0.001** |  | **-0.55 (-0.80 to -0.30)** | **<0.001** |
| DAS28-P | **-0.22 (-0.26 to -0.19)** | **<0.001** |  | **-0.26 (-0.35 to -0.17)** | **<0.001** |  | **-0.41 (-0.50 to -0.33)** | **<0.001** |  | **-0.43 (-0.68 to -0.19)** | **0.001** |
| Tender-swollen difference | **-0.14 (-0.18 to -0.11)** | **<0.001** |  | **-0.16 (-0.23 to -0.08)** | **<0.001** |  | **-0.20 (-0.30 to -0.11)** | **<0.001** |  | **-0.39 (-0.63 to -0.16)** | **0.002** |
| Tender:swollen ratio | **-0.07 (-0.10 to -0.03)** | **<0.001** |  | **-0.15 (-0.23 to -0.06)** | **0.001** |  | **-0.10 (-0.19 to -0.02)** | **0.019** |  | -0.21 (-0.51 to 0.09) | 0.170 |

Linear regression of z-transformed variables, adjusted for inflammation (ln-transformed CRP. Regression coefficients (95% CI) and p values for associations with pain measures. Negative B values represent variables associating with worse pain, as SF36-Bodily Pain is a negative index and lower pressure pain thresholds (PPT) indicate greater sensitivity. TJC=tender joint count, SJC=swollen joint count, ESR=erythrocyte sedimentation rate, VAS-GH=visual analogue scale-general health. DAS28-P=proportion of DAS28-ESR attributable to patient-reported components. No corrects were performed for multiple comparisons. Statistically significant findings highlighted in **bold**.

Figure S1: Measurement properties of each index


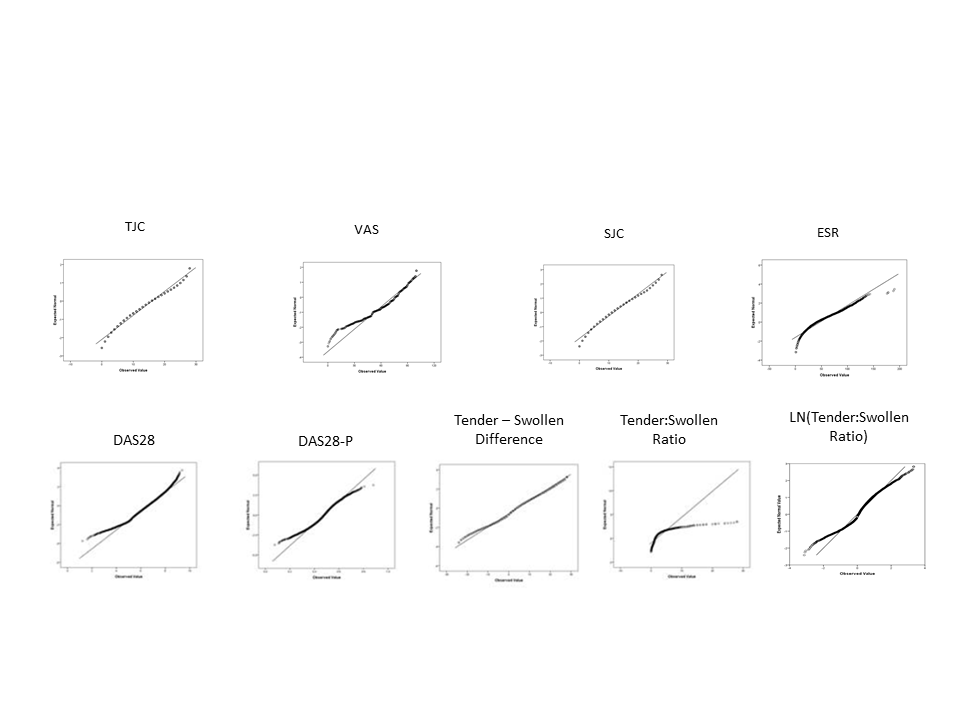


Q-Q plots for each index taken at baseline in the BSRBR- anti-TNF cohort. Tender:Swollen ratio and LN(Tender:Swollen ratio) are both included for comparison. DAS28-P=proportion of DAS28-ESR attributable to patient-reported components.
